# Based on SEER Database: Population Distribution, Survival Analysis, and Prognostic Factors of Organ Metastasis of Lung Large Cell Neuroendocrine Carcinoma

**DOI:** 10.3389/fonc.2022.810170

**Published:** 2022-03-18

**Authors:** Chang-fu Liu, Yu-jian Tao

**Affiliations:** ^1^ Medical College, Yangzhou University, Yangzhou, China; ^2^ Department of Respiratory and Critical Care Medicine, The Affiliated Hospital of Yangzhou University, Yangzhou University, Yangzhou, China

**Keywords:** lung large cell neuroendocrine carcinoma, organ metastasis, survival analysis, prognostic factors, population distribution

## Abstract

**Background:**

The incidence rate of lung large cell neuroendocrine carcinoma (LCNEC) in lung cancer is low, but the malignancy is high and the prognosis is poor. We used the Surveillance, Epidemiology, and End Results (SEER) database to determine the population distribution of organ metastasis in LCNEC, conduct survival analysis, judge prognostic factors, and provide direction for follow-up diagnosis and treatment.

**Materials and methods:**

By logging into the SEER database, the data of lung LCNEC were retrieved and the target population was selected. According to the presence or absence of organ metastasis (bone, brain, liver, and lung), we divided the target population into the no organ metastasis group (n = 1,202) and the organ metastasis group (n = 870). By analyzing the clinicopathological data of patients and using the survival function, the corresponding median survival time was obtained, and the influencing factors of each group were analyzed. Then, the significant influencing factors were analyzed by multivariate Cox regression analysis to screen out the independent influencing factors.

**Result:**

In the overall sample group, multivariate Cox regression analysis showed that sex, age, primary site surgery, bone metastasis, brain metastasis, liver metastasis, radiotherapy, and chemotherapy were independent prognostic factors. The 1-year survival rate was 13.8% in the bone metastasis group, 19.1% in the brain metastasis group, 13.8% in the liver metastasis group, and 20.3% in the intrapulmonary metastasis group. In the organ metastasis group, multivariate Cox regression analysis showed that sex, chemotherapy, radiotherapy sequence with surgery, primary site surgery, liver metastasis, and age at diagnosis were independent factors affecting the prognosis.

**Conclusion:**

In the overall sample of LCNEC, bone metastasis, brain metastasis, and liver metastasis all reduced the overall survival time, while the effect of intrapulmonary metastasis on the overall survival time was not statistically significant. Sex, chemotherapy, radiotherapy sequence with surgery, primary site surgery, liver metastasis, and age were independent factors affecting the prognosis of the LCNEC organ metastasis group. Women, chemotherapy, and radiotherapy sequence with surgery were favorable factors, while old age, liver metastasis, and male were unfavorable factors.

## Introduction

Lung large cell neuroendocrine carcinoma (LCNEC) is a neuroendocrine tumor. Its biological behavior is similar to that of small cell lung cancer, but it belongs to a variant of large cell carcinoma (1). The content of LCNEC in the 5th edition of WHO thoracic tumor classification in 2021 has little change from that in 2015; LCNEC is defined as a tumor with cytological characteristics of non-small cell lung cancer (abundant cytoplasm, large cells, and prominent nucleoli), NE morphology (nested organoids with trabeculae, palisade, and rosette), and high proliferation. The mitotic rate is more than 10 mitotic images per 2 mm^2^, and at least one of the three markers (synaptophysin, chromogranin A, and CD56) was positive (2, 3). It is a rare disease, accounting for about 3% of lung cancer (4). However, it has high malignancy and poor prognosis compared with other non-small cell lung cancer (5). Once organ metastasis occurs, the survival time is only a few months. Therefore, in order to understand the clinicopathological features and prognostic factors of lung LCNEC after organ metastasis, and then provide guidance for clinical treatment, we conducted relevant specific research.

## Materials and Methods

Apply for an account at www.seer.cancer.gov; download the SEER * Stat version 8.3.9.2 software (**Table 1** for detail); open it through file; select the database of incidence-SEER 18 Regs Custom Data (with additional treatment fields); click Edit; select {site and morphology.site recode ICD-O-3/WHO 2008} = ‘lung and bronchus’, and {race, sex, year DX, registry, county, year of diagnosis} = ‘2010’, ‘2011’, ‘2012’, ‘2013’, ‘2014’, ‘2015’; select the factors of Race, Sex, Site recode ICD-O-3/WHO 2008, Primary Site, Histologic Type ICD-O-3, Grade, Laterality, T, N, M, RX Summ–Surg Prim Site (1998+), Radiation sequence with surgery, Radiation recode, Chemotherapy recode, SEER Combined Mets at DX-brain/bone/liver/lung, CS tumor size, Survival months, Vital status recode (study cutoff used), Age at diagnosis, Insurance Recode, Marital status at diagnosis, and Patient ID. Then 309,696 cases were selected, and 8,013 (lung LCNEC number) were selected by histologic type ICD-O-3. Then, 2,199 cases remained; 2,198 cases remained after unknown factors were removed from survival months. A total of 2,108 cases remained after C34.0-main bronchus was removed from the primary site label. Then remove “unknown” in all four groups of SEER combined Mets at DX brain (bone, live, lung) (2010 +), leaving 2,072 cases. According to the presence or absence of organ metastasis (bone, brain, liver, and lung), we divided the cases with no metastasis of four organs into the no organ metastasis group (n = 1,202) and those with one or more organ metastasis into the organ metastasis group (n = 870). By analyzing the clinicopathological data of patients and using the survival function, we obtained the corresponding median survival time and analyzed the influencing factors of each group. Then the significant influencing factors were analyzed by multivariate Cox regression, so as to screen out the independent influencing factors.

**Table 1:** SEER data acquisition steps. A total of 2,072 cases were included in the study analysis.

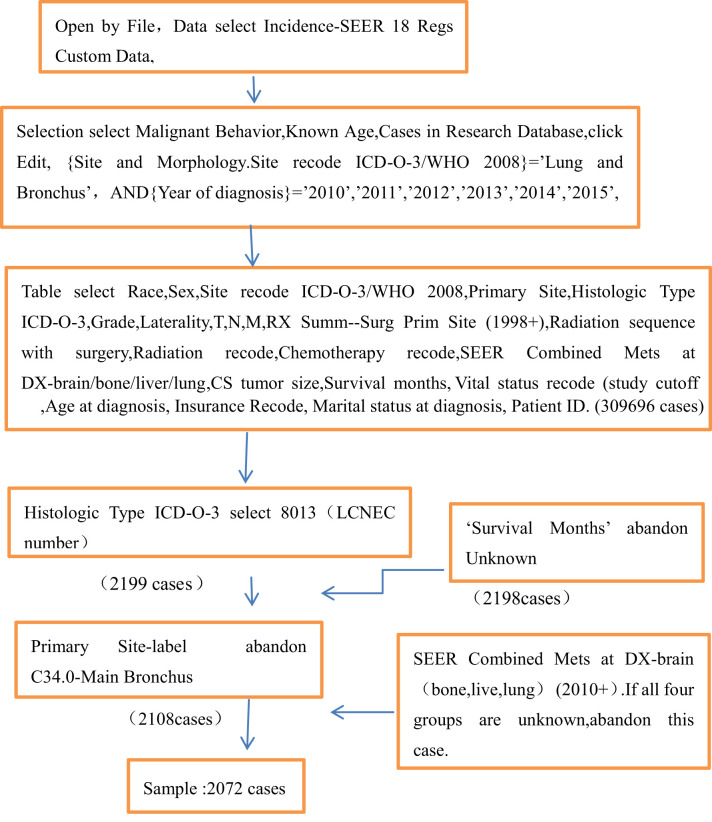

### Inclusion Criteria

(1) The location of the primary cancer is in the lung, (2) the period of diagnosis is 2010–2015, and (3) the code of the histological type of lung cancer is 8013 (lung LCNEC number).

### Exclusion Criteria

(1) The primary site is located in the bronchus, (2) the follow-up survival status is unclear, and (3) the four common metastatic sites are unclear.

### Grouping Method

A total of 2,072 cases were included. According to the presence or absence of organ metastasis (bone, brain, liver, and lung), we formed the cases without metastasis of four organs into the no organ metastasis group (n = 1,202) and the cases with one or more organ metastasis into the organ metastasis group (n = 870). This study has been reviewed by the ethics committee of our hospital.

### Statistical Method

The clinicopathological data of patients were analyzed by the SPSS21 software. The basic pathological data of each group were analyzed by cross-table chi-square test. *P* < 0.05 showed a significant difference. The survival curve was drawn one by one by using the survival function Kaplan–Meier to obtain the median survival time. Log rank (mental Cox) was used to test the significant difference. The factors with *P* < 0.05 were the factors affecting the prognosis. The factors with significant differences were analyzed by multivariate Cox regression analysis.

## Result

### Population Distribution

Based on the analysis of the basic pathological data of 2,072 cases, it can be seen that the proportion of men in the two groups is higher than that of women, of which 57.7% are men in the organ metastasis group. The proportion of people over 60 years old is significantly higher than that of people under 60 years old, of which 72.2% are over 60 years old in the organ metastasis group. In the race comparison, whites are significantly higher than other races, of which 85.1% are whites in the organ metastasis group. The proportion of death cases in both groups was high, of which the death in the organ metastasis group accounted for 94.8%. In the organ metastasis group, according to the frequency of metastasis, the order from high to low is liver, brain, bone, and lung. The most common metastasis of two organs is liver + bone (**Table 2** for Details).

**Table 2:** Basic clinicopathological features.

Factor	Classification	No organ metastasis group n = 1,202 (%)	Organ metastasis group n = 870 (%)	X^2^/f	*P*
Age	Less than 60 years old	282(23.5)	242(27.8)	5.067	*P*=0.024
	60 years or older	920(76.5)	628(72.2)		
Race	White	994(82.7)	740(85.1)	2.949	*P*=0.229
	Black	160(13.3)	94(10.8)		
	Other	48(4)	36(4.1)		
Sex	Female	574(47.8)	368(42.3)	6.057	*P*=0.014
	Male	628(52.2)	502(57.7)		
Grade	Grade I	4(0.3)	4(0.5)	213.731	*P<*0.001
	Grade II	19(1.6)	3(0.3)		
	Grade III	545(45.3)	178(20.5)		
	Grade IV	166(13.8)	71(8.2)		
	Unknown	468(38.9)	614(70.6)		
Laterality	Left—origin of primary	487(40.5)	319(36.7)	54.659	*P<*0.001
	Right—origin of primary	689(57.3)	477(54.8)		
	Paired site	25(2.1)	43(4.9)		
	Unknown	1(0.1)	31(3.6)		
T	T0	8(0.7)	9(1.0)	233.591	*P<*0.001
	T1	396(32.9)	110(12.6)		
	T2	417(34.7)	236(27.1)		
	T3	70(5.8)	25(2.9)		
	T4	262(21.8)	372(42.8)		
	Tx	49(4.1)	118(13.6)		
N	N0	639(53.2)	203(23.3)	220.064	*P<*0.001
	N1	121(10.1)	73(8.4)		
	N2	314(26.1)	382(43.9)		
	N3	109(9.1)	165(19)		
	Nx	19(1.6)	47(5.4)		
M	M0	1,029(85.6)	13(1.5)	1,432.102	*P<*0.001
	M1	168(14)	851(97.8)		
	Mx	5(0.4)	6(0.7)		
Primary site surgery	No	535(44.5)	825(94.8)	566.564	*P<*0.001
	Yes	667(55.5)	45(5.2)		
Radiotherapy + surgery	Yes	174(14.5)	165(19)	7.434	*P=*0.006
	No	1,028(85.5)	705(81)		
Chemotherapy	Yes	578(48.11)	492(56.6)	14.482	*P<*0.001
	No	624(51.9)	378(43.4)		
Tumor size (mm)	0	9(0.7)	9(0.7)	54.887	*P<*0.001
	Less than 10	21(1.7)	9(1)		
	10–988	1,063(88.4)	676(77.7)		
	Other	109(9.1)	176(20.2)		
survival time	Less than 12 months	435(36.2)	727(83.6)	481.600	*P<*0.001
	More than 12 months	339(28.2)	97(11.1)		
	More than 24 months	150(12.5)	30(3.4)		
	More than 36 months	109(12.5)	11(1.3)		
	More than 48 months	62(9.1)	3(0.3)		
	More than 60 months	107(5.2)	2(0.2)		
Survival state	Dead	788(65.6)	825(94.8)	250.736	*P<*0.001
	Alive	414(34.4)	45(5.2)		
Insurance	Any Medicaid	166(13.8)	142(16.3)	10.841	*P*=0.013
	Unknown	21(1.7)	10(1.1)		
	Uninsured	22(1.8)	32(3.7)		
	Insured	993(82.6)	686(78.91)		
Marital status	Divorced	175(14.6)	110(12.6)	11.616	*P*=0.04
	Married	603(50.2)	455(52.3)		
	Widowed	178(14.8)	98(11.3)		
	Unknown	60(5)	38(4.4)		
	Single	166(13.8)	152(17.5)		
	Separated	20(1.7)	17(2)		

It can be seen that the proportion of men in the two groups is higher than that of women, of which 57.7% are men in the organ transfer group, and the proportion of people over 60 years old is significantly higher than that of people under 60 years old, of which 72.2% are over 60 years old in the organ transfer group. In the racial comparison, whites are significantly higher than other races, of which 85.1% are whites in the organ metastasis group. The proportion of death cases in the two groups was high, of which the death in the organ metastasis group accounted for 94.8%.

### Related Prognostic Factors

**Figure 1:**
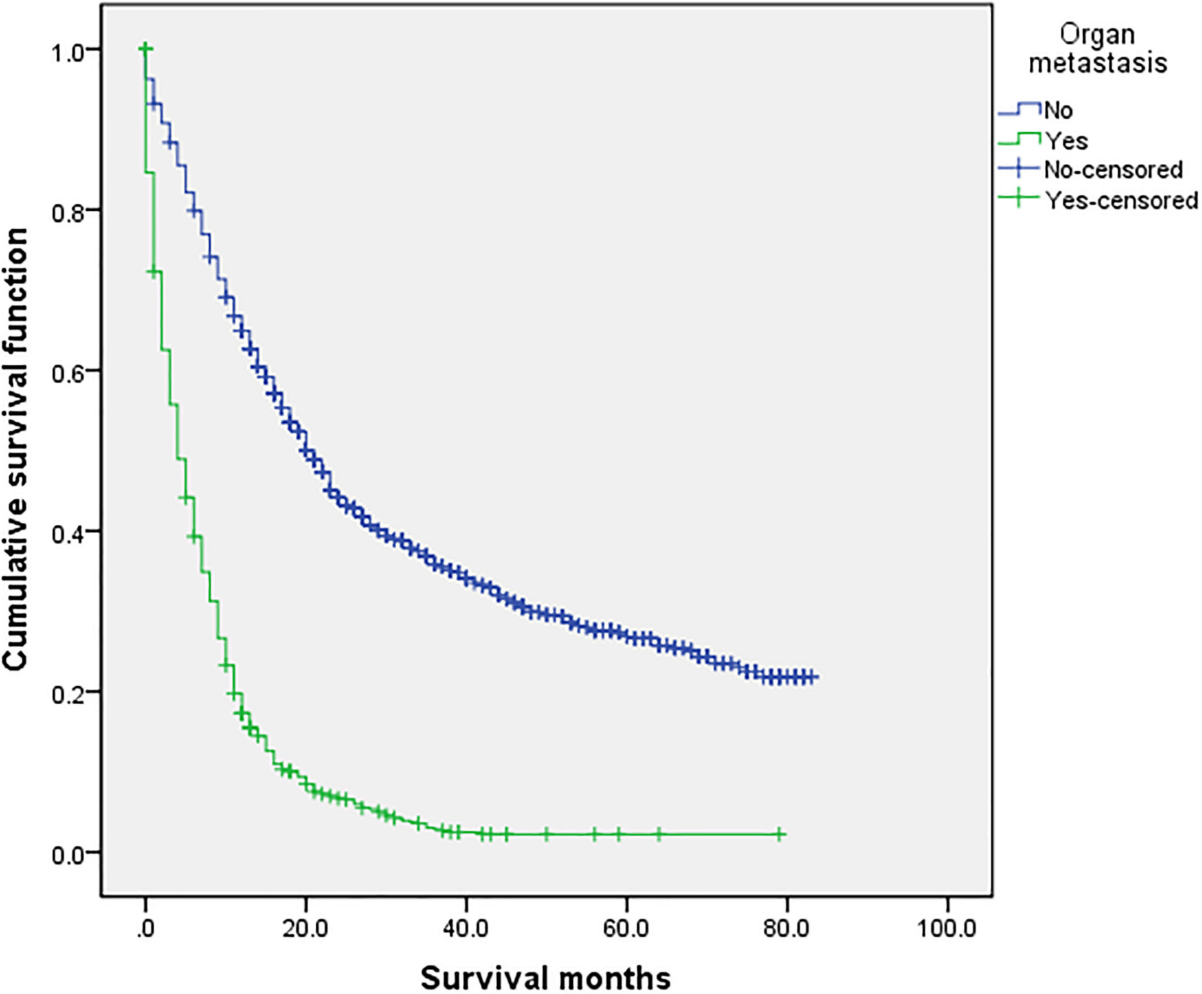
Comparison of survival time between the two groups. In the overall sample (n = 2,072), univariate analysis of the no organ metastasis group and the organ metastasis group showed that P < 0.001 was obtained by Kaplan–Meier survival analysis log rank (mental Cox). There was significant difference between the two groups.

**Figure 2:**
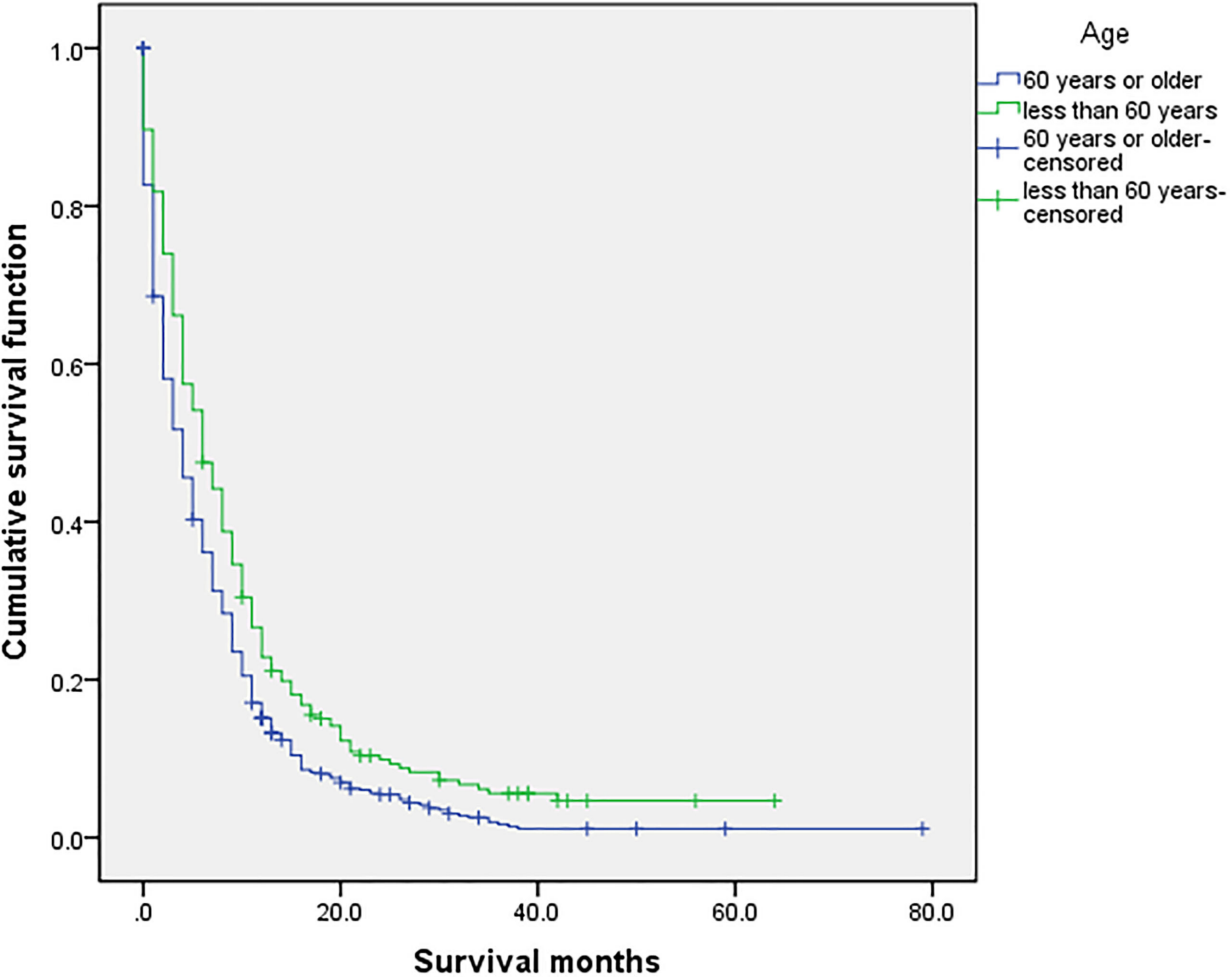
Univariate analysis of 60 years or older and less than 60 years. In the organ metastasis group (n = 870), the univariate analysis of 60 years or older and less than 60 years showed that *P <*0.001 was obtained by Kaplan–Meier survival analysis log rank (mental Cox). There was significant difference between the two groups.

**Figure 3:**
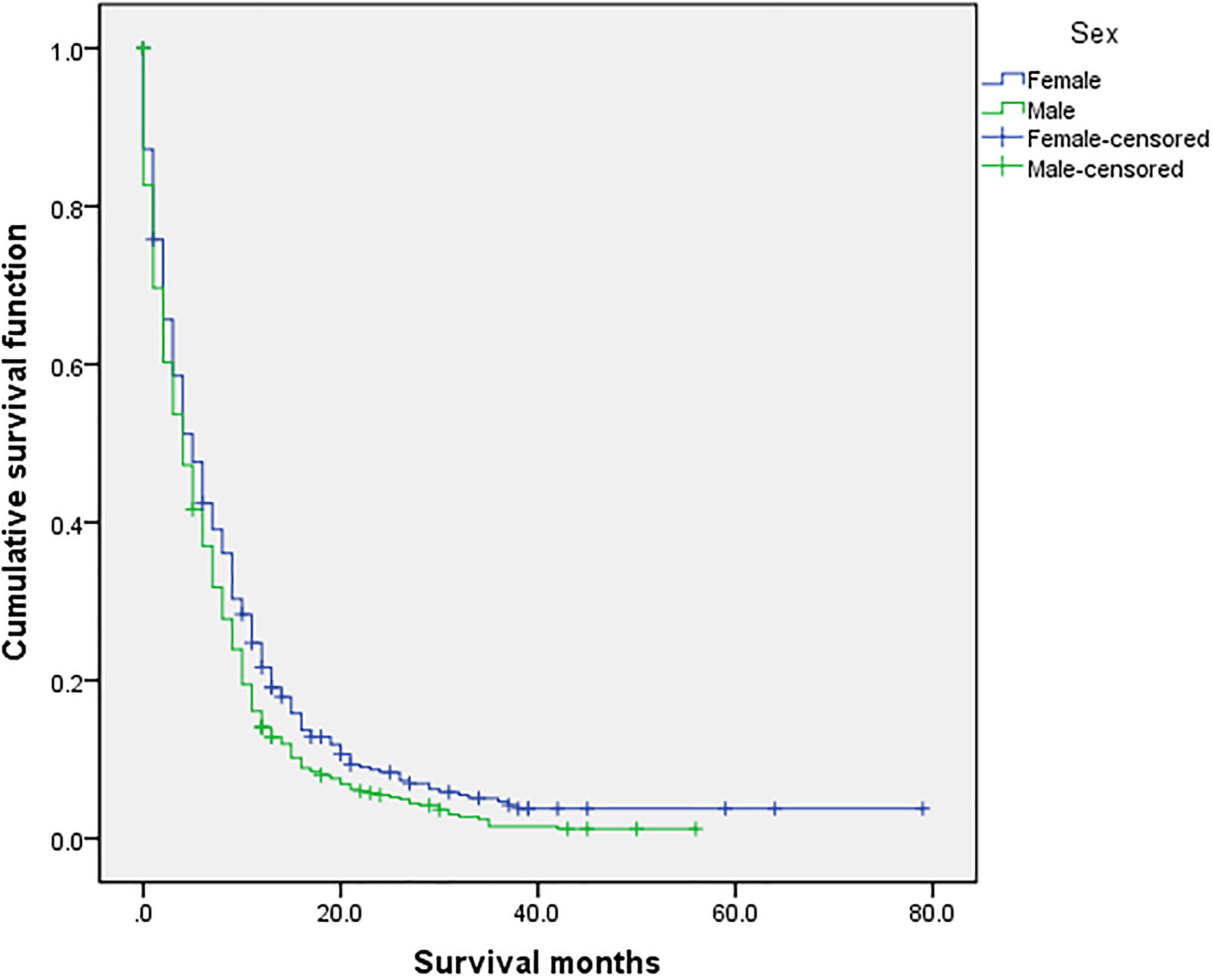
Univariate analysis of female and male. In the organ metastasis group (n = 870), univariate analysis of female and male showed that *P* =0.003 was obtained by Kaplan–Meier survival analysis log rank (mental Cox). There was significant difference between the two groups.

**Figure 4:**
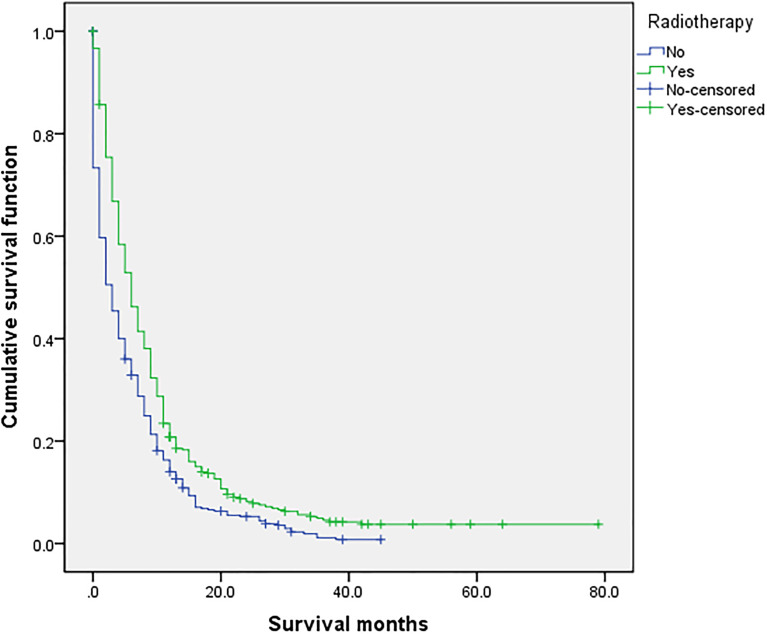
Univariate analysis of with or without radiotherapy. In the organ metastasis group (n = 870), univariate analysis of with or without radiotherapy showed that *P* < 0.001 was obtained by Kaplan–Meier survival analysis log rank (mental Cox). There was significant difference between the two groups.

**Figure 5:**
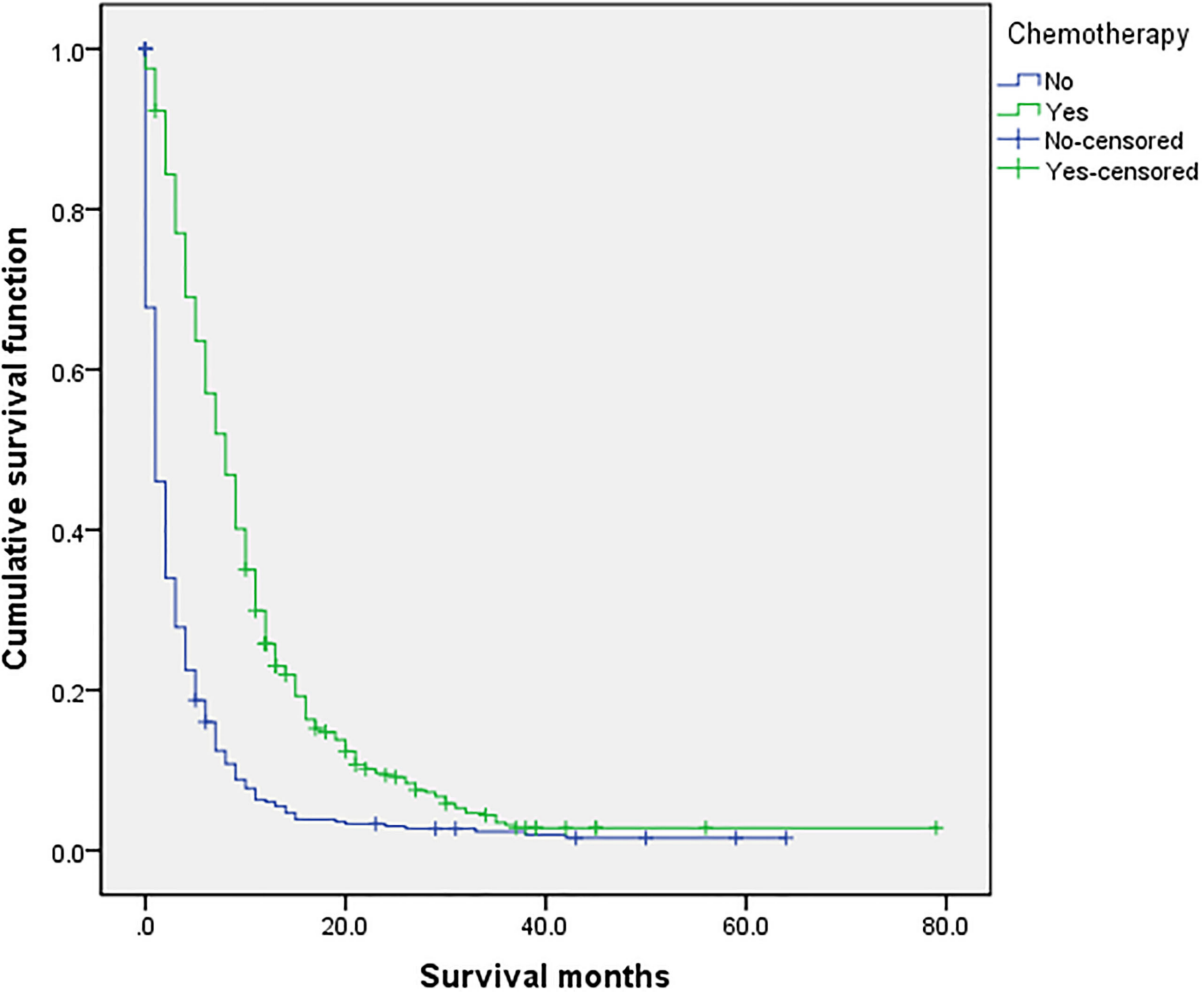
Univariate analysis of with or without chemotherapy. In the organ metastasis group (n = 870), univariate analysis of with or without chemotherapy showed that P < 0.001 was obtained by Kaplan–Meier survival analysis log rank (mental Cox). There was significant difference between the two groups.

**Figure 6:**
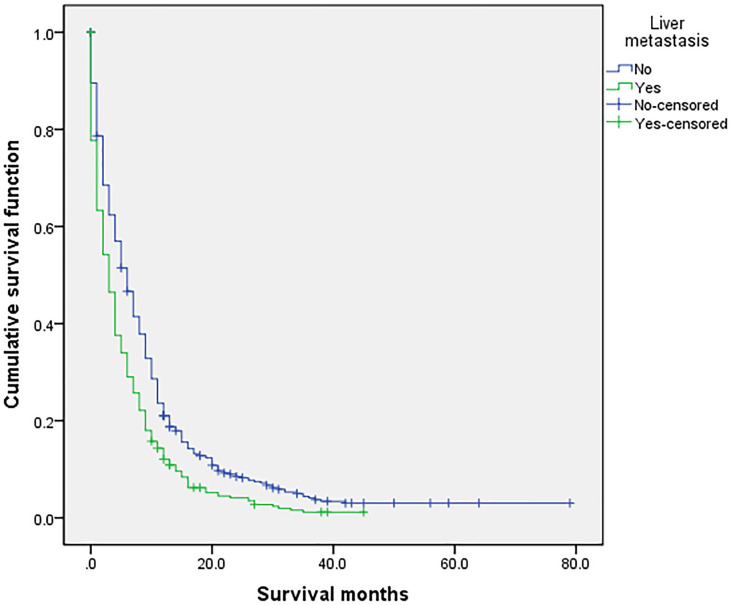
Univariate analysis of liver metastasis. In the organ metastasis group (n = 870), univariate analysis of liver metastasis and no liver metastasis showed that *P* < 0.001 was obtained by Kaplan–Meier survival analysis log rank (mental Cox). There was significant difference between the two groups.

Use the survival function to draw a survival curve for each factor in turn, and log rank (mental Cox) test for significant difference. Factors with *P* < 0.05 are the prognostic factors (**Table 3**). In the overall sample, sex (*P* 0.000), primary site (*P* 0.000), histological grade (*P* 0.000), laterality (*P* 0.000), T (*P* 0.000), N (*P* 0.000), M (*P* 0.000), primary site surgery (*P* 0.000), radiotherapy (*P* 0.000), insurance (*P* 0.008), organ metastasis (*P* 0.000) (**Figure 1**), liver metastasis (*P* 0.000), brain metastasis (*P* 0.000), bone metastasis (*P* 0.000), intrapulmonary metastasis (*P* 0.000), and age at diagnosis (*P* 0.000) were all factors affecting survival time. The *P* value of chemotherapy was 0.055, close to 0.05. We carried out multivariate Cox regression analysis with the factors listed in **Table 4**. The results showed that age (P 0.000 HR 1.241, 95%CI 1.104–1.396), sex (P 0.000 HR 0.793, 95%CI 0.717–0.877), primary site surgery (P 0.000 HR 2.473, 95%CI 2.094–2.920), radiotherapy (P 0.000 HR 1.250, 95%CI 1.109–1.409), chemotherapy (P 0.000 HR 2.267, 95%CI 2.025–2.539), bone metastasis (P 0.009 HR 0.828, 95%CI 0.718–0.954), brain metastasis (P 0.000 HR 0.720, 95%CI 0.619–0.837), and liver metastasis (P 0.000 HR 0.666, 95%CI 0.578–0.767) were all independent factors affecting the prognosis. A total of 870 patients with organ metastasis were analyzed for prognostic factors. By Kaplan–Meier’s analysis, age (*P* 0.000) (**Figure 2**), sex (*P* 0.003) (**Figure 3**), T (*P* 0.002), N (*P* 0.005), primary site surgery (*P* 0.000), radiotherapy (*P* 0.000) (**Figure 4**), chemotherapy (*P* 0.000) (**Figure 5**), bone metastasis (*P* 0.01), and liver metastasis (*P* 0.000) (**Figure 6**) were all factors affecting survival time. Multivariate Cox regression analysis showed that sex (P 0.000 HR 0.774, 95%CI 0.673–0.891), radiotherapy sequence with surgery (P 0.032 HR 1.234, 95%CI 1.018–1.496), chemotherapy (p 0.000 HR 2.866, 95%CI 2.466–3.331), age (P 0.025 HR 1.200, 95%CI 1.024–1.407), primary site surgery (P 0.018 HR 1.500, 95%CI 1.071–2.103) and liver metastasis (P 0.000 HR 0.683, 95%CI 0.590–0.790) were all independent factors affecting the prognosis.

**Table 3:** Univariate Cox analysis (overall and organ metastasis group).

	Overall (n = 2072)		Organ metastasis group (n = 870)	
Factor	X^2^/f	*P* value	X^2^/f	*P* value
Organ metastasis	669.482	*P<*0.001	–	–
Sex	30.833	*P<*0.001	8.614	*P*=0.003
Race	2.748	*P=0.253*	1.180	*P*=0.554
Primary site	89.510	*P<*0.001	4.720	*P*=0.317
Grade	129.045	*P<*0.001	4.661	*P*=0.324
Laterality	14.506	*P*=0.002	3.365	*P*=0.339
T	345.769	*P<*0.001	18.45	*P*=0.002
N	419.014	*P<*0.001	15.007	*P*=0.005
M	682.722	*P<*0.001	3.531	*P*=0.171
Primary site surgery	603.674	*P<*0.001	24.512	*P<*0.001
Radiotherapy + surgery	0.847	*P*=0.357	29.495	*P<*0.001
Radiotherapy	20.858	*P<*0.001	33.700	*P<*0.001
Chemotherapy	3.692	*P*=0.055	188.354	*P<*0.001
Bone metastasis	269.658	*P<*0.001	7.536	*P*=0.01
Brain metastasis	168.078	*P<*0.001	4.739	*P*=0.059
Liver metastasis	392.654	*P<*0.001	32.561	*P<*0.001
Lung metastasis	141.120	*P<*0.001	1.381	*P*=0.699
Insurance	9.701	*P*=0.008	3.724	*P*=0.155
Marital status Age	8.654 9.66	*P*=0.124 *P*=0.002	10.027 17.28	*P*=0.074 *P<*0.001

In the overall sample, sex (P 0.000), primary site (P 0.000), histological grade (P 0.000), laterality (P 0.000), T (P 0.000), N (P 0.000), M (P 0.000), primary site surgery (P 0.000), radiotherapy (P 0.000), insurance (P 0.008), organ metastasis (P 0.000), liver metastasis (P 0.000), brain metastasis (P 0.000), bone metastasis (P 0.000), intrapulmonary metastasis (P 0.000), and age (P 0.002) were all factors affecting survival time.

A total of 870 patients with organ metastasis were analyzed for prognostic factors. By Kaplan–Meier analysis, sex (P 0.003), T (P 0.002), N (P 0.005), primary site surgery (P 0.000), radiotherapy (P 0.000), chemotherapy (P 0.000), age (P 0.000), bone metastasis (P 0.01), and liver metastasis (P 0.000) were all factors affecting survival time.

**Table 4:** Multivariate Cox analysis (organ metastasis group and the overall sample).

Group	Factor	B	SE	*P*	HR	95%CI
Organ metastasis group (n=870)	Sex	-0.256	0.072	0.000	0.774	0.673-0.891
	Age	0.183	0.081	0.025	1.200	1.024-1.407
	Chemotherapy	1.053	0.077	0.000	2.866	2.466-3.331
	Radiotherapy+surgery	0.210	0.098	0.032	1.234	1.018-1.496
	Primary site surgery	0.406	0.172	0.018	1.500	1.071-2.103
	Liver metastasis	-0.382	0.074	0.000	0.683	0.590-0.790
Overall (n=2072)	Age	0.216	0.060	0.000	1.241	1.104-1.396
	Sex	-0.232	0.051	0.000	0.793	0.717-0.877
	Primary site surgery	0.905	0.085	0.000	2.473	2.094-2.920
	Radiotherapy	0.223	0.061	0.000	1.250	1.109-1.409
	Chemotherapy	0.819	0.058	0.000	2.267	2.025-2.539
	Bone metastasis	-0.189	0.073	0.009	0.828	0.718-0.954
	Brain metastasis	-0.329	0.077	0.000	0.720	0.619-0.837
	Liver metastasis	-0.406	0.072	0.000	0.666	0.578-0.767

Organ metastasis group: Multivariate cox regression analysis showed that sex (P 0.000 HR 0.774, 95%CI 0.673–0.891), radiotherapy +surgery (P 0.032 HR 1.234, 95%CI 1.018–1.496), chemotherapy (p 0.000 HR 2.866, 95%CI 2.466–3.331), age (P 0.025 HR 1.200, 95%CI 1.024–1.407), primary site surgery (P 0.018 HR 1.500, 95%CI 1.071–2.103), and liver metastasis (P 0.000 HR 0.683, 95%CI 0.590–0.790) were all independent factors affecting the prognosis.

The overall sample: multivariate Cox regression analysis showed that age (P 0.000 HR 1.241, 95%CI 1.104–1.396), sex (P 0.000 HR 0.793, 95%CI 0.717–0.877), primary site surgery (P 0.000 HR 2.473, 95%CI 2.094–2.920), radiotherapy (P 0.000 HR 1.250, 95%CI 1.109–1.409), chemotherapy (p 0.000 HR 2.267, 95%CI 2.025–2.539), bone metastasis (P 0.009 HR 0.828, 95%CI 0.718–0.954), brain metastasis (P 0.000 HR 0.720, 95%CI 0.619–0.837), and liver metastasis (P 0.000 HR 0.666, 95%CI 0.578–0.767) were all independent factors affecting the prognosis.

### Survival Analysis

The overall median survival time of the disease was 10 months (**Table 6**), 21 months in the no organ metastasis group, 4 months in the organ metastasis group, 4 months in the bone metastasis and lung metastasis groups, 3 months in the liver metastasis group, and 5 months in the brain metastasis group. In the no organ metastasis group (n = 1,202), the median survival time was 17 months for men (**Table 7**), 25 months for women, 26 months for patients under the age of 60, and 20 months for patients over the age of 60. In the organ metastasis group (n = 870), the median survival time was 4 months for men, 5 months for women, 6 months for patients under the age of 60, and 4 months for patients over the age of 60. In the comparison of survival time between the two groups with or without organ metastasis (**Table 5**), the 1-year survival rate was 63.8% in the no organ metastasis group, 16.4% in the organ metastasis group, 13.8% in the bone metastasis group, 19.1% in the brain metastasis group, 13.8% in the liver metastasis group, and 20.3% in the intrapulmonary metastasis group. The 3-year survival rate was 23.1% in the no organ metastasis group, 1.9% in the organ metastasis group, 0.6% in the bone metastasis group, 3.1% in the brain metastasis group, 0.8% in the liver metastasis group, and 1.8% in the intrapulmonary metastasis group. The 5-year survival rate was 8.9% in the no organ metastasis group, 0.2% in the organ metastasis group, 0.6% in the brain metastasis group, and zero in the other metastasis groups (**Tables 5**–**7** for Details).

**Table 5:** Survival time comparison.

Survival Months	No organ metastasis group	Organ metastasis group	Bone metastasis	Brain metastasis	Liver metastasis	Lung metastasis
More than 12 months	63.8%	16.4%	13.8%	19.1%	13.8%	20.3%
More than 24 months	35.6%	5.3%	3.9%	6.3%	3.3%	4.8%
More than 36 months	23.1%	1.9%	0.6%	3.1%	0.8%	1.8%
More than 48 months	14%	0.6%	0	1.4%	0	0.4%
More than 60 months	8.9%	0.2%	0	0.6%	0	0

As shown in Table 5, The 1-year survival rate in the non organ metastasis group was 63.8%, while that in the organ metastasis group was only 16.4%, bone metastasis group was 13.8%, brain metastasis group was 19.1%, liver metastasis group was 13.8% and intrapulmonary metastasis group was 20.3%, The 3-year survival rate in the non organ metastasis group was 23.1%, while that in the organ metastasis group was only 1.9%, bone metastasis group was 0.6%, brain metastasis group was 3.1%, liver metastasis group was 0.8% and intrapulmonary metastasis group was 1.8%,The 5-year survival rate in the non organ metastasis group was 8.9%, while that in the organ metastasis group was only 0.2%, brain metastasis group was 0.6%, The other metastasis groups were 0.

**Table 6:** Median survival months.

The whole sample (n = 2,072)							
Factors	Overall	Organ metastasis	No organ metastasis	Bone metastasis	Brain metastasis	Liver metastasis	Lung metastasis
Median survival months	10	4	21	4	5	3	4

The overall median survival time of the disease was 10 months, 21 months in the non-organ metastasis group, 4 months in the organ metastasis group, 4 months in the bone metastasis and lung metastasis groups, 3 months in the liver metastasis group, and 5 months in the brain metastasis group.

**Table 7:** Median survival time comparison.

Factor	Classification	No organ metastasis group Median survival time (months)	Organ metastasis group Median survival time (months)
Age	Less than 60 years old	26	6
	60 years or older	20	4
Race	White	22	4
	Black	19	4
	Other	15	5
Sex	Female	25	5
	Male	17	4
Grade	Grade I	46	8
	Grade II	53	11
	Grade III	23	5
	Grade IV	25	5
	Unknown	16	4
Laterality	Left—origin of primary	4	4
	Right—origin of primary	4	4
	Paired site	7	7
	Unknown	4	4
T	T0	15	15
	T1	6	6
	T2	5	5
	T3	2	2
	T4	4	4
	Tx	4	4
N	N0	6	6
	N1	6	6
	N2	4	4
	N3	4	4
	Nx	4	4
M	M0	3	3
	M1	4	4
	Mx	5	5
Primary site surgery	No	4	4
	Yes	9	9
Radiotherapy	Yes	6	6
	No	3	3
Chemotherapy	Yes	8	8
	No	1	1
Insurance	Insured	4	4
	Unknown	4	3
	Uninsured	3	4
Marital status	Divorced	4	4
	Married	5	5
	Widowed	3	3
	Unknown	4	4
	Single	4	4
	Separated	4	4

In the no organ metastasis group (n = 1,202): the median survival time was 17 months for men, 25 months for women, 26 months for patients under the age of 60, and 20 months for patients over the age of 60.

In the organ metastasis group (n = 870): the median survival time was 4 months for men, 5 months for women, 6 months for patients under the age of 60, and 4 months for patients over the age of 60.

## Discussion

In the overall sample of LCNEC, bone metastasis, brain metastasis, and liver metastasis all reduced the overall survival time, while the effect of intrapulmonary metastasis on the overall survival time was not statistically significant. In the organ metastasis group, the 1-year survival rate of lung LCNEC after organ metastasis was only 16.4%. The 3-year survival rate was only 1.9%. The median survival time was only 4 months. The median survival time of liver metastasis was the shortest: only 3 months. Sex, chemotherapy, radiotherapy sequence with surgery, primary site surgery, liver metastasis, and age were independent factors affecting the prognosis of the LCNEC organ metastasis group. Women, chemotherapy, and radiotherapy sequence with surgery were favorable factors, while old age, liver metastasis, and male were unfavorable factors.

Lung LCNEC is a rare disease with high malignancy and poor prognosis. In order to master its basic clinical characteristics and the impact of related treatment on its prognosis, we use the American SEER database established in 1973. Many cases of LCNEC were recorded in this database. We selected 2,072 cases (including 870 cases of organ metastasis) for study and analyzed their basic clinicopathological data. It was found that age, sex, primary site surgery, radiotherapy, chemotherapy, bone metastasis, brain metastasis, and liver metastasis were independent factors affecting the prognosis of LCNEC. The independent factors affecting the prognosis of the LCNEC organ metastasis group were sex, chemotherapy, radiotherapy sequence with surgery, primary site surgery, liver metastasis, and age. More men are suffering from LCNEC than women, and the survival time of men is significantly shorter than that of women. In the whole sample, the median survival time is 9 months for men and 13 months for women. In the organ metastasis group, the median survival time is 4 months for men and 5 months for women. The older the age, the more patients with LCNEC, and the survival time of people over 60 years old is significantly shorter than that of people under 60 years old. In the whole sample, the median survival time is 12 months for people under the age of 60 and 10 months for people over the age of 60. In the organ metastasis group, the median survival time is 6 months for people under the age of 60 and 4 months for people over the age of 60. According to the analysis, it may be related to male smoking and the length of smoking time, since smoking is the main risk factor of lung cancer (6). Nasim F and other researchers indicated that the intensity of smoking is proportional to the risk of disease (7). LCNEC itself is refractory (8); the survival time is very short after organ metastasis. The multivariate Cox regression analysis of the organ metastasis group showed that chemotherapy and radiotherapy sequence with surgery can prolong the survival time and improve the prognosis. At present, there is no standard treatment guideline for LCNEC. The commonly used chemotherapy schemes are as follows: one is the SCLC scheme of platinum combined with etoposide, and the other is the NSCLC scheme of platinum combined with gemcitabine and pemetrexed (9). Hiroshima K’s studies showed that LCNEC is a group of tumors with biological and histological heterogeneity. Surgical treatment is recommended for early LCNEC. Combined with platinum etoposide chemotherapy, it can prevent the recurrence of surgically resected LCNEC (10).Other studies have shown that when the expression of DLL3 in LCNEC patients is negative, the application of platinum containing chemotherapy can improve the survival time of patients, and DLL3 can be used as a sensitive index for monitoring chemotherapy (11). Some scholars showed that platinum-based combined chemotherapy has a short-term effect when LCNEC metastases happen. At present, there is no second-line choice. Radiotherapy and immunotherapy have a synergistic effect, but the best combination method is not clear (12). With the development of medicine, some researchers use genomic typing to select different chemotherapy drugs to improve the prognosis (6). Researchers such as ODA R found that immune checkpoint inhibitors combined with cytotoxic anticancer drugs after radiotherapy are effective for LCNEC patients with distant multiple metastases (13). Wegner RE and other researchers indicated that radiotherapy for LCNEC with brain metastasis can improve the survival time (14). All studies have confirmed that chemotherapy and radiotherapy sequence with surgery are independent influencing factors to improve the prognosis of LCNEC. In this study, liver metastasis is an independent prognostic factor for organ metastasis of LCNEC. In order to study its treatment, the final IMpower 150 survival analysis was conducted, which showed that ACP (atzozumab + carboplatin + paclitaxel) had a quantitative advantage in the overall survival rate compared with BCP (bevacizumab + carboplatin + paclitaxel). However, it was not statistically significant. In the 20 months of follow-up, compared with BCP, ABCP (atzozumab + bevacizumab + carboplatin + paclitaxel) can continuously improve the overall survival time of patients in all patients (including patients sensitized by EGFR mutation, patients with liver metastasis, and patients who failed previous tyrosine kinase inhibitor treatment) (15, 16). This study has several limitations. First, a small number of cases in the SEER database still have incomplete and uncertain registration, which is particularly evident in the metastasis of various organs. The reason may be due to the short survival time of patients after diagnosis, lack of time for relevant CT examination, or the patients’ refusal of the examination. Second, the study shows that chemotherapy and radiotherapy sequence with surgery are independent factors to improve the prognosis of the LCNEC organ metastasis group, but the drug use of chemotherapy is unclear, the length of drug use is not recorded, and the specific time of radiotherapy is not recorded. Third, in order to facilitate the study of etiology, occupational and exposure factors of patients can be added to the database. In conclusion, the incidence rate of LCNEC in the middle and old age is high, the prognosis is poor, and the median survival time is short after distant organ metastasis. At this time, radiotherapy sequence with surgery and chemotherapy can prolong the survival time and improve prognosis.

## Data Availability Statement

The original contributions presented in the study are included in the article/supplementary material. Further inquiries can be directed to the corresponding author.

## Author Contributions

C-fL: resources, data curation, software, formal analysis, funding acquisition, writing—original draft, and project administration. Y-jT: validation and writing—review and editing. All authors contributed to the article and approved the submitted version.

## Conflict of Interest

The authors declare that the research was conducted in the absence of any commercial or financial relationships that could be construed as a potential conflict of interest.

## Publisher’s Note

All claims expressed in this article are solely those of the authors and do not necessarily represent those of their affiliated organizations, or those of the publisher, the editors and the reviewers. Any product that may be evaluated in this article, or claim that may be made by its manufacturer, is not guaranteed or endorsed by the publisher.
